# The Abdominal Circulatory Pump

**DOI:** 10.1371/journal.pone.0005550

**Published:** 2009-05-14

**Authors:** Andrea Aliverti, Dario Bovio, Irene Fullin, Raffaele L. Dellacà, Antonella Lo Mauro, Antonio Pedotti, Peter T. Macklem

**Affiliations:** 1 Dipartimento di Bioingegneria, Politecnico di Milano, Milan, Italy; 2 INRCA Hospital, Casatenovo (Lc), Italy; 3 Meakins-Christie Laboratories, Royal Victoria Hospital, McGill University Health Centre Research Institute, Montreal, Quebec, Canada; The Research Center of Neurobiology - Neurophysiology of Marseille, France

## Abstract

Blood in the splanchnic vasculature can be transferred to the extremities. We quantified such blood shifts in normal subjects by measuring trunk volume by optoelectronic plethysmography, simultaneously with changes in body volume by whole body plethysmography during contractions of the diaphragm and abdominal muscles. Trunk volume changes with blood shifts, but body volume does not so that the blood volume shifted between trunk and extremities (Vbs) is the difference between changes in trunk and body volume. This is so because both trunk and body volume change identically with breathing and gas expansion or compression. During tidal breathing Vbs was 50–75 ml with an ejection fraction of 4–6% and an output of 750–1500 ml/min. Step increases in abdominal pressure resulted in rapid emptying presumably from the liver with a time constant of 0.61±0.1SE sec. followed by slower flow from non-hepatic viscera. The filling time constant was 0.57±0.09SE sec. Splanchnic emptying shifted up to 650 ml blood. With emptying, the increased hepatic vein flow increases the blood pressure at its entry into the inferior vena cava (IVC) and abolishes the pressure gradient producing flow between the femoral vein and the IVC inducing blood pooling in the legs. The findings are important for exercise because the larger the Vbs the greater the perfusion of locomotor muscles. During asystolic cardiac arrest we calculate that appropriate timing of abdominal compression could produce an output of 6 L/min. so that the abdominal circulatory pump might act as an auxiliary heart.

## Introduction

We have previously reported that in normal subjects exercising with experimentally induced expiratory flow-limitation there were blood shifts from the trunk to the extremities [1]. These amounted to about 300 ml, or ∼70 ml per kPa alveolar pressure. The blood shifts were calculated by integrating flow at the mouth to obtain expired and inspired volumes simultaneously with the volume change of the trunk during breathing measured by optoelectronic plethysmography [OEP, 2] (see Appendix S1 for a complete glossary of symbols). During exercise without flow-limitation these two measurements of volume were identical using this technique. During expiration when expiratory flow was limited, the volume change of the trunk led the volume exhaled at the mouth in time and was of greater amplitude. We thought this was due to gas compression in the lung. To verify this we estimated alveolar pressure from esophageal pressure, and because we knew absolute gas volume in the lung from our OEP measurements we calculated the volume of compressed gas. To our surprise this accounted for less than half of the greater volume displacement of the trunk.

We reasoned that trunk volume could only change for three reasons during the course of our experiment: gas leaving and entering at the mouth, gas compression and expansion in the lung, and blood shifts between the trunk and the extremities. As we measured the first and calculated the second we attributed the remainder to blood shifts. These were associated with high expiratory pressures in both the pleural and abdominal cavities which acted like a Valsalva maneuver and decreased cardiac output by ∼10% [3].

We thought that these findings might provide a clue as to the mechanism by which coughing during asystolic cardiac arrest can maintain consciousness in human subjects [4], [5]. But in order to study blood shifts accurately we needed to: 1) develop a method to measure the blood shifts and the amount of gas compression and decompression more precisely; 2) avoid the effects of high pleural pressures on the cardiac chambers, pulmonary blood volume and venous return.

In the present experiments we used whole body plethysmography (WBP) which measures changes in body volume (ΔVb), to quantify the volume of air flowing in and out of the lungs, and the volume of gas compression or decompression. WBP is insensitive to blood shifts whereas OEP measures the same variables as WBP plus any blood shifts between the trunk and the extremities. Thus under conditions when changes in pulmonary or splanchnic blood volume and in the size of the great vessels in the thoracic and abdominal cavities are so small as to be negligible, the measured changes in body and trunk volume should be virtually identical.

By combining OEP with WBP measured simultaneously, ΔVb and changes in trunk volume (ΔVtr) were measured continuously. The parameters causing ΔVb can be expressed as:
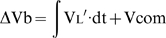
(1)where ∫VL'·dt is the integral of flow at the mouth and Vcom is the volume of gas compressed or decompressed. In the presence of blood shifts between the trunk and the extremities:
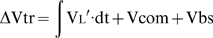
(2)where Vbs is the volume of blood shifts. Subtracting equation 1 from equation 2 yields:
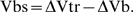
(3)By making simultaneous measurements of ΔVtr and ΔVb we were able to measure Vbs continuously as a function of time and by differentiation, obtain flow. We refer to this technique as double plethysmography.

To avoid the effects of high pleural pressure we studied expulsive maneuvers performed by simultaneously contracting the diaphragm and abdominal muscles thereby increasing abdominal pressure (Pab) while keeping pleural pressure (Ppl) limited to the changes during quiet breathing. This removed the effects of high Ppl on venous return, pulmonary blood volume and on the cardiac chambers. However the high Pab may well have affected inferior vena caval flow and this is discussed in detail later. Because pulmonary blood volume presumably only changed to the extent that it varies with quiet breathing, the only other source of significant blood shifts, is the splanchnic blood reservoir which contains ∼1.3 L or 20–25% of the normal blood volume [6], [7]. Thus we assumed that the source of the blood shifts we measured was the splanchnic vascular bed.

In the present communication we report the difference between ΔVb as measured by WBP and ΔVtr as measured by OEP during expulsive maneuvers of various sorts and during quiet breathing using different patterns of breathing. We found that increasing abdominal pressure displaced substantial quantities of blood from the trunk to the extremities. This may have important implications for cardio-pulmonary resuscitation and exercise performance.

## Results

**Figure 1:**
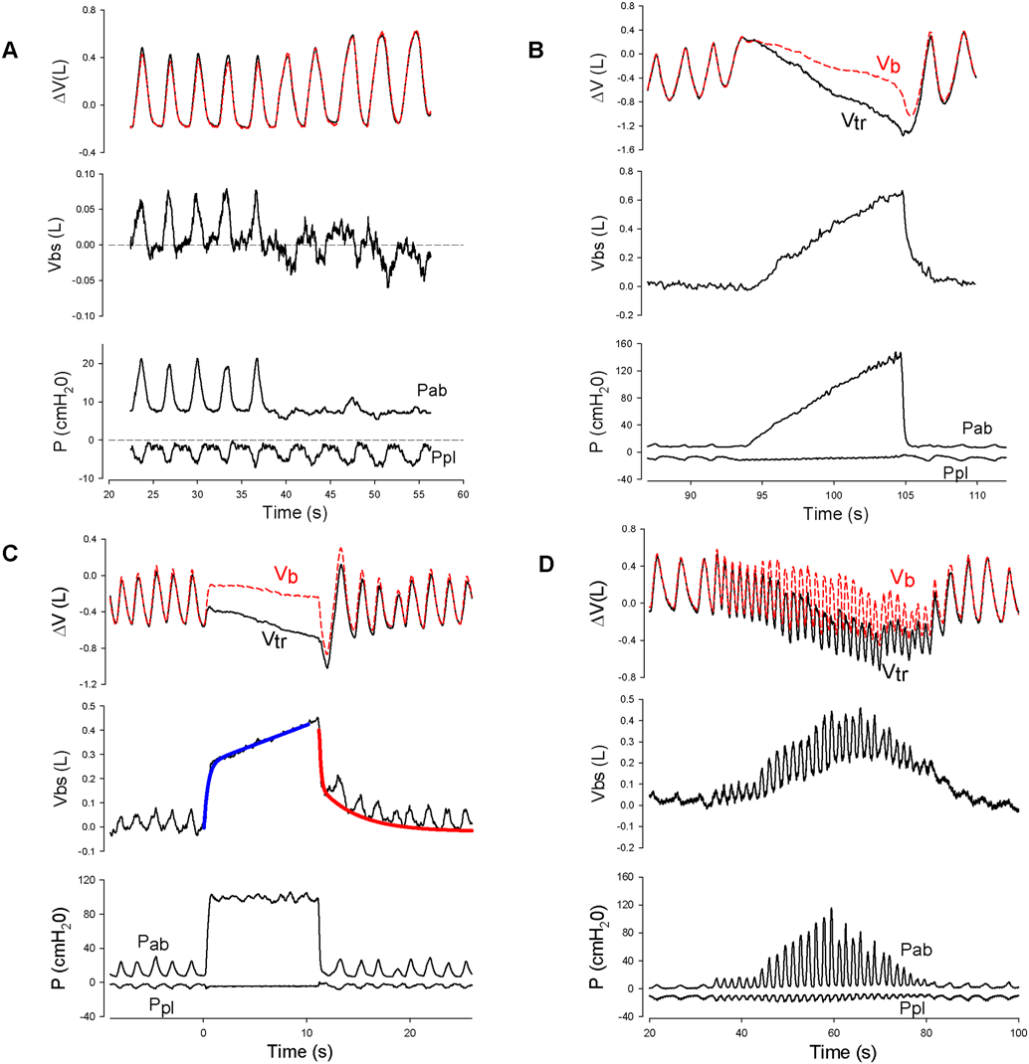
Recordings of pressures and volumes during respiratory maneuvers. 1-A. Tracings during spontaneous quiet breathing and minimization of inspiratory increases in abdominal pressure (Pab). Top tracings: changes in body volume (Vb, red dashed line) and the volume of the trunk (Vtr, black solid line) measured simultaneously showing the ventilatory pattern. The difference between Vb and Vtr gives the volume of blood shifted from the splanchnic vascular bed to the extremities (Vbs) shown in the middle tracing. Blood shifts (60–75 ml) from the splanchnic bed are in phase with ΔPab during spontaneous breathing but when ΔPab was minimized, the blood shifts abruptly changed both phase and magnitude. Bottom traces: abdominal pressure (Pab) and pleural pressure (Ppl). 1-B. Simultaneous recordings of Vb (red dashed line) and Vtr (black solid line) (top tracings) during a ramp increase in Pab while Ppl remained unchanged (bottom tracings). The volume of blood shifted from the splanchnic vascular bed to the extremities (Vbs) is shown in the middle tracing. 1-C. Effect of a step increase in Pab (bottom trace) on Vbs shown in the top and middle traces. There was an initial rapid shift in blood probably from the liver followed by a slower one probably from the non-hepatic splanchnic vasculature. We were able to fit the emptying curve with a double exponential shown by the blue solid line. Refilling was modeled as a single exponential (red solid line). From these exponentials time constants for filling and emptying were calculated (table 1). 1-D. Tracings during rapid breathing with progressive breath-by-breath increases in Pab followed by progressive decreases as shown in the bottom traces. During the period of rapid breathing there were equally rapid blood shifts coming from the fast compartment. As the amplitude of the Pga swings increased, blood shifts of ∼350 ml came from the slow space. This space refilled as the amplitude of the Pga swings decreased. Strong and sustained increases in Pga can transfer a significant fraction of the splanchnic blood reservoir to perfuse locomotor muscles and other body tissues in a physiologic form of blood doping.

During tidal breathing Vbs was 50–75 ml (figure 1-A). Assuming a splanchnic blood volume of ∼1.3 L [6], [7] this gives an ejection fraction of 4–6% and with a respiratory frequency of 15–20 breaths/min, an output of 750–1500 ml/min (15–30% of cardiac output). Figure 1-B illustrates a ramp increase in Pab. The difference between the Vb and Vtr traces is Vbs. In this instance when Pab reached 140 cm H_2_O (14kPa) more than 600 ml of blood was displaced.

Figure 1-C shows a step increase in Pab, held constant for 10 seconds, followed by a step decrease. The Vbs response is shown, both for splanchnic blood emptying during the step increase and refilling following the step decrease. The time course of Vbs during emptying was fitted with a double exponential shown by the blue line while filling was fitted with a single exponential shown by the red line. The responses to step changes were used to calculate the time constants for filling and emptying shown in table 1. During step increases in Pab splanchnic blood emptying came first from a rapid compartment well characterized by a single exponential, followed by a compartment which emptied much more slowly. Refilling was well described by a single exponential for the whole splanchnic vascular bed with a time constant similar to that of the rapid emptying time compartment (0.57±0.23 sec r^2^ = .0.971 vs 0.61±0.22 sec, r^2^ = .983, respectively).

**Table 1:** Time constants for splanchnic emptying and refilling

	Emptying			Refilling	
Subject	τ_1_ (sec)	τ_2_ (sec)	r^2^	τ_1_ (sec)	r^2^
# 1	0.49	17.21	0.998	0.44	0.994
# 2	0.48	>100	0.978	0.73	0.987
# 3	0.93	>100	0.977	0.91	0.988
# 4	0.40	>100	0.986	0.40	0.981
# 5	0.73	>100	0.978	0.31	0.965
# 6	-	-	-	0.63	0.909
Mean	0.61	>100	0.983	0.57	0.971
SD	0.22	-	0.010	0.23	0.032

Figure 1-A is an example of Vbs with normal increases in Pab during quiet breathing and during rib cage breaths with little change in Pab. The immediate marked reduction in Vbs with rib cage breathing indicates that fluctuations in Pab are responsible for the blood shifts.

Figure 1-D is an example of breathing more rapidly with diaphragmatic descent and contraction of abdominal muscles during expiration. This tracing illustrates the rapid blood shifts nearly in phase with Pab and the slower progressive increase and subsequent decrease in Vbs over time. These data show that sufficiently rapid breaths can prevent complete refilling in the time available during inspiration, so that part of Vbs remains in the extremities.

**Figure 2:**
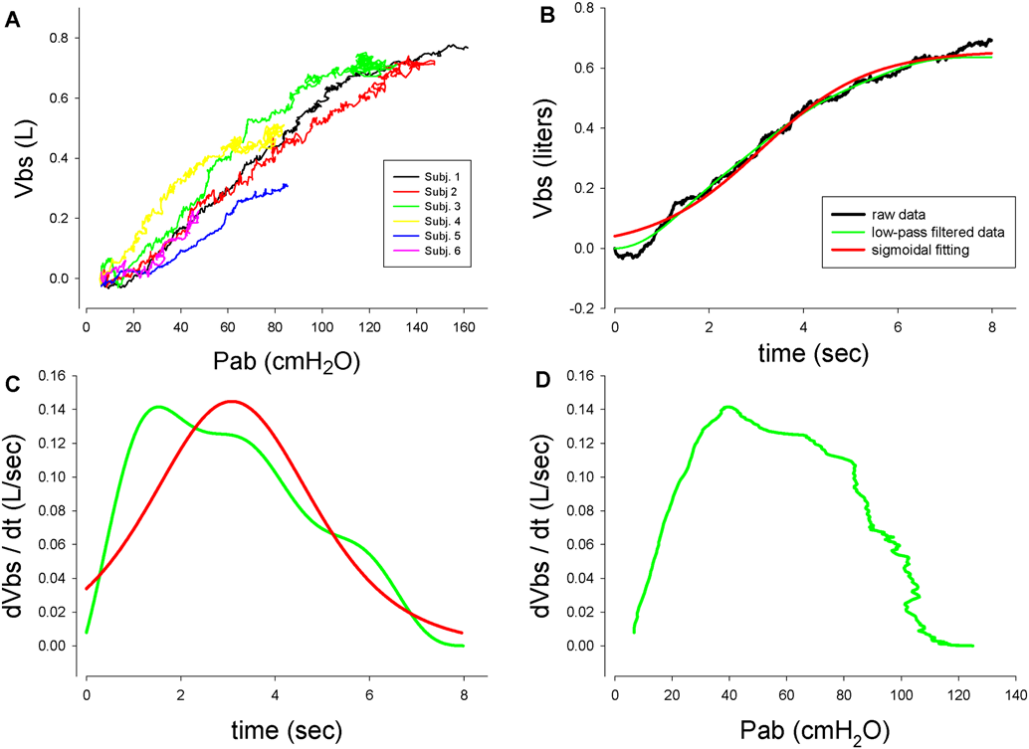
Pressure, volume and flow relationships. 2-A. Volume of blood shifts during ramp increases in Pab in all subjects. Each colour represents a single subject. 2-B. A representative tracing of Vbs during a ramp maneuver as a function of time showing a typical sigmoid shape. The red line superimposed on the raw data results from low-pass filtering and the green line from curve fitting with a sigmoid. 2-C. Flow from the splanchnic bed (dVbs/dt) obtained by differentiating the green and red lines. Both curves indicate that flow increases, reaches a maximum and then decreases nearly to zero. 2-D. The Pab-flow curve shows an initially rapidly increasing flow, a maximum and then a decrease as Pab increases, falling to zero at high Pab. This is explained by the mechanics of fluid flow in a collapsible tube described in the text.

Figure 2-A, shows Pab vs Vbs plots during ramp increases in Pab in the 6 subjects in whom we made these curves. The slopes give the volume of blood displaced from the splanchnic bed per unit increase in Pab under quasi-static conditions which averaged ∼5.5 ml/cm H_2_O (55ml/kPa) Pab in good agreement with previous estimates using much cruder measurements of Vbs [1]. These slopes are not estimates of splanchnic vascular compliance because splanchnic intravascular pressure was not measured,

Figure 2-B shows a typical sigmoid-shaped Vbs vs time plot . We differentiated this to obtain splanchnic outflow (Fig. 2-C) and plotted it against Pab (Fig. 2-D). Three distinct Pab splanchnic blood flow regimes are shown: 1) as Pab increased splanchnic flow increased; 2) with further increases in Pab splanchnic flow decreased; 3) at the highest value of Pab, splanchnic flow became zero.

## Discussion

### Main Findings

During ramp increases in Pab there were monotonically increasing displacements of blood from the trunk presumably coming from the splanchnic vascular bed. By differentiation of the volume signal we observed that as Pab increased, flow initially increased, reached a maximal value and then decreased and became zero at the highest value of Pab. Assuming that when flow was zero, all the stressed volume of the splanchnic bed had been expelled, and that the total splanchnic blood volume was ∼1.3 L [7], [8], the stressed volume is 40–50% of total splanchnic blood volume. During step increases in Pab, splanchnic blood emptied first from a rapid compartment followed by a compartment which emptied much more slowly. Refilling was well described by a single exponential for the whole splanchnic vascular bed. If breathing became sufficiently rapid there was insufficient time for complete refilling of the splanchnic vasculature so that the increase in blood volume of the extremities lasted as long as the time constant for refilling was long relative to the time available.

Most previous investigations of the mechanisms by which splanchnic blood can be recruited have focused on reflex changes in splanchnic vascular tone, in particular the role of the splanchnic veins and venules [8]–[10] . To our knowledge, the present report is the first systematic investigation of the role of abdominal pressure in displacing splanchnic blood.

### Critique of Methods

**Figure 3:**
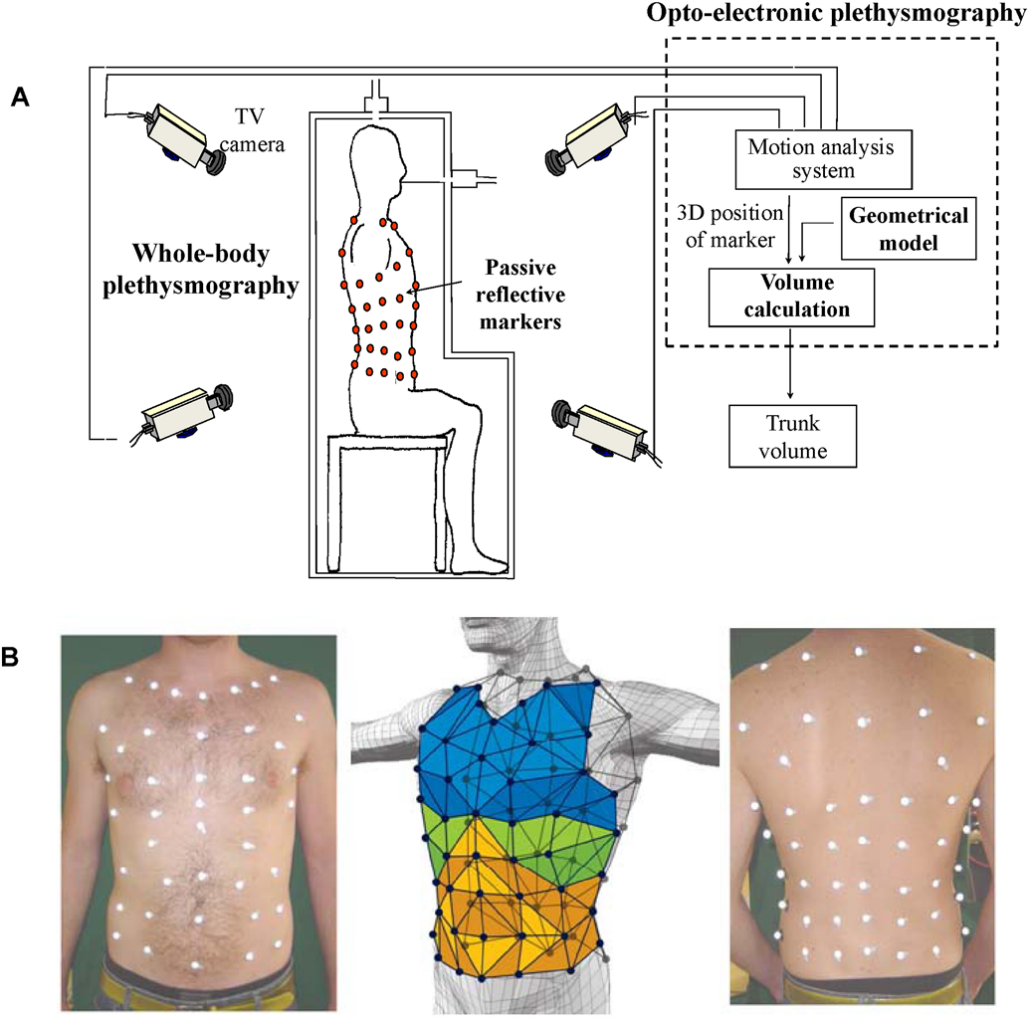
Experimental set-up. 3-A Schematic diagram of the experimental set-up used to measure Vbs as the difference between changes in body volume and trunk volume (ΔVb-ΔVtr). ΔVb and ΔVtr are measured simultaneously by whole body plethysmography and Opto-electronic plethysmography (OEP), respectively. OEP is based on a motion analysis system which measures the 3D positions of 89 reflective markers positioned on body surface by a set of TV cameras, and computes the volume of the trunk by Gauss's Theorem. 3-B Marker positions on the anterior (left photograph) and posterior (right photograph) trunk surface. In the middle figure a diagram of the thoraco-abdominal surface triangulation is shown.

The use of OEP to measure ΔVtr requires tracking of 89 reflective markers attached to the surface of the chest wall ventrally and dorsally, by video cameras as illustrated in figure 3. The measurement of ΔVtr has been extensively validated [1], [2]. What is new in the present research is that the markers were detected by video cameras outside a transparent WBP while the subject was inside. Thus the light rays between the markers and the video cameras were subject to refraction as they passed through the walls of the body box. To correct for this potential source of error a bar containing markers of known distance between them was moved inside the box to calibrate the system and thereby correct for any refraction error. To make sure that this potential source of error was eliminated we estimated the volume of solids whose true volume was known. The error of the estimates was always <±0.3%. When blood shifts are minimal, to a close approximation ΔVtr = ΔVb. Indeed during quiet breathing the ΔVtr and ΔVb tracings were virtually superimposable as illustrated in figure 1-A. This could only be the case if Vtr were correctly measured, because in the absence of blood shifts or changes in gas volume of the upper airway including the cheeks, body volume only changes to the extent that trunk volume changes. Thus using WBP as a gold standard, we found our measurements of ΔVtr by OEP to be remarkably accurate. From this we infer that the measurements of the volume of blood shifts were also accurate.

At high gain we showed small blood shifts of 60–70 ml during quiet breathing as shown in figure 1-A. We were concerned that small differences in sensitivity between OEP and WBP could account for these differences during quiet breathing which were often only 10% of the tidal volume signal. If this had been the case, the phase of Vbs would always bear the same relationship to the phases of ΔVb and ΔVtr. But this was not what was observed. When diaphragmatic breathing was changed to rib cage breathing the phase of Vbs changed according to the phase of Pab, not Vb and Vtr (fig 1-A). Similarly when abdominal muscle contraction occurred either voluntarily or during quiet breathing, the phase of Vbs became biphasic, in phase with the biphasic increases in Pab during both inspiration and expiration. Therefore the blood shifts we measured during quiet breathing could not have resulted from minor calibration differences between the OEP and WBP signals. Furthermore, when breathing took place with only minimal fluctuations in Pab we found, as predicted, that the blood shifts were very small and the WBP and OEP tracings were essentially identical. Figure 1-A shows this to be the case. The small fluctuations ranging between +20 and −50 ml approximately180° out of phase with Ppl swings are easily explained by small fluctuations in intrathoracic volume resulting from the changes in pleural pressure.

We measured blood shifts during expulsive maneuvers while Ppl remained unchanged from quiet breathing. By avoiding the rise in Ppl we also avoided the influence of Valsalva maneuvers on venous return and the effects of a high Ppl on the cardiac chambers and pulmonary blood volume. Because the major pressure change took place only in the abdominal cavity, fluctuations in pulmonary blood volume should be small and similar to those during quiet breathing. The major source of the measured blood shifts then, should be from the abdominal contents. This conclusion is supported by Takata et al [11] who found that the increase in abdominal pressure was more important than the fall in pleural pressure during diaphragmatic contraction under hypervolemic conditions so that central venous pressure rose, rather than falling during inspiration.

While we attributed these shifts to changes in splanchnic blood volume, some of the blood leaving the abdominal cavity could have been due to reductions in the volume of blood within the inferior vena cava or the abdominal aorta. We doubt that the inferior vena cava became markedly compressed during the expulsive maneuvers. The hemodynamics of flow through collapsible vessels which are distended at the upstream end but compressed downstream (discussed in detail below) lead to a pressure drop along the collapsible vessel due to frictional resistance and convective acceleration so that an increasing external pressure acting on the vessel (in this instance Pab) always becomes greater than the lateral internal pressure at the downstream extremity of the vessel (in this instance just rostral to the point where the inferior vena cava passes through the diaphragm Thus dynamic compression starts at this point. Assuming that the dimensions and mechanical properties of the inferior vena cava are independent of its length, the dynamic compression should be limited to the short distance between the entry of the hepatic vein into the vena cava and where the cava enters the diaphragm. Flow would not stop, because if it did the pressure at the point of compression would equilibrate with the upstream pressure and the vena cava would reopen. Thus the inferior vena caval compression in the abdomen would only occur at its downstream extremity and this could only contribute a very small fraction to the blood shift volumes we measured.

Although we did not monitor blood pressure during the course of these experiments we believe that it was unlikely that the abdominal aorta was subjected to the same sort of dynamic compression that the inferior vena cava experienced. Pab rarely, if ever, exceeded the normal systolic blood pressure. But, presumably there was an oscillatory variation in aortic cross-sectional area between systole and diastole. Indeed the small rapid oscillations of 10–15 ml with a frequency of ∼80/min which can be seen in fig. 1-A might well be the result of pulsatile abdominal aortic dimensions. If so it is unlikely that the aorta contributed significantly to the blood shifts we measured. For these reasons we believe that our assumptions that the blood shifts came from the splanchnic vascular bed are justified.

For the purposes of our analysis we assume that abdominal pressure changes were everywhere equal based upon Duomarco and Rimini's model of the abdomen as a liquid-filled sac [12], Nevertheless Decramer et al found considerable variation in regional abdominal pressure swings when different pressures were applied over different regions [13]. Thus, Brienza et al [14] postulated that subdiaphragmatic pressure acting on the liver might compress hepatic sinusoids and increase the resistance to venous flow through the liver. They measured small but progressive increases in hepatic sinusoidal and venous resistance as positive end-expiratory pressure (PEEP) increased. PEEP of 5, 10 and 15 cmH_2_O increased portal venous pressure by an amount equal to the increases in right atrial pressure, namely by 1.3, 2.7 and 4.8 mm Hg respectively. With a hepatic vascular compliance, estimated to be 71.4ml*kPa^−1^ (vide infra: section headed Effects of Splanchnic Outflow on Cardiac Output) this would result in increases in hepatic vascular volume of 91, 189 and 336 ml respectively Such increases in volume might amplify the subdiaphragmatic pressure acting on the liver so that the hepatic sinusoidal resistances increased progressively in response to diaphragmatic contraction. Indeed a plot of the increase in resistance they measured vs the increase in portal venous pressure extrapolates very close to a zero increase in resistance when there is no PEEP. Furthermore Decramer et al found no significant difference in abdominal pressure swings measured in different locations during quiet breathing in dogs [13]. Thus in our subjects in the absence of PEEP we doubt that diaphragmatic compression of normovolemic hepatic sinusoids significantly increased the resistance to venous flow through the liver.

### Splanchnic Vascular Dynamics

**Figure 4:**
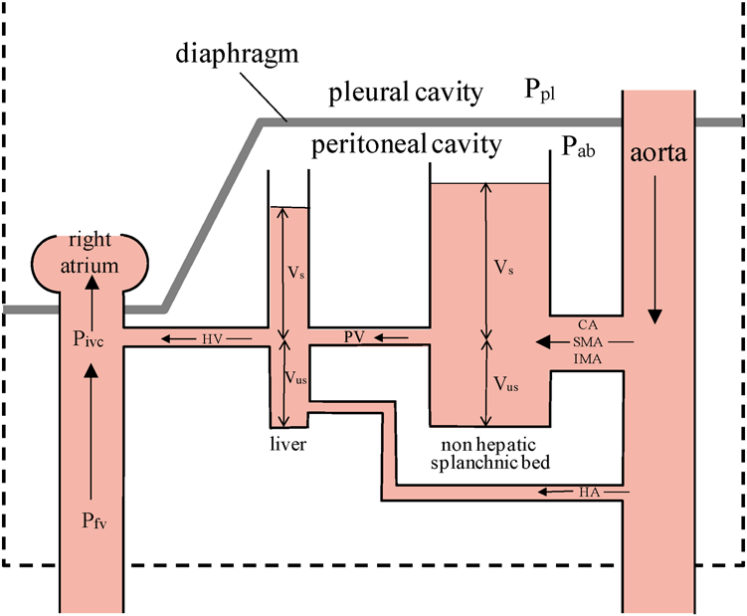
Hydraulic model of the splanchnic vascular bed, consisting of two capacitors, the liver vasculature and the non-hepatic splanchnic vascular bed. CA = celiac artery; SMA = superior mesenteric artery; IMA = inferior mesenteric artery; HA = hepatic artery; Vs = stressed volume of blod where vascular transmural pressure >0; Vus = unstressed volume where vascular transmural pressure <0; Ppl = pleural pressure; Pab = abdominal pressure; PV = portal vein; HV = hepatic vein; Pfv = pressure in the femoral vein; Pivc = pressure in the inferior vena cava at the entry of the hepatic vein. The height of the column of blood indicates that there is a pressure gradient from aorta > mean vascular pressure in the non hepatic vessels > mean vascular pressure in the hepatic vascular bed > right atrial pressure.

Figure 4 is a hydraulic model of the splanchnic vascular bed, which we use to explain our findings. Blood flows directly into the non-hepatic splanchnic vascular bed through the celiac, and the superior and inferior mesenteric arteries and into the liver through the hepatic artery; but most of the liver's blood supply comes from the portal vein which drains nearly all of the non-hepatic vascular bed. In order for the blood to flow from the non-hepatic viscera through the liver to the right atrium, there must be a pressure gradient. Measurements of these pressures in the rabbit showed that the pressure in the portal vein was ∼7.7 mm Hg (1kPa), in the hepatic sinusoids, ∼5.4 mm Hg (0.6kPa) and in the inferior vena cava close to the entry of the hepatic vein, ∼3.9 mm Hg (0.5kPa) higher than the pressure in the middle of the right atrium [15]. The splanchnic venous blood empties via the hepatic vein into the inferior vena cava immediately below the diaphragm.

In the model the non-hepatic splanchnic vascular bed and the liver vasculature are represented as two capacitors. The capacitance, or the volume of blood contained at a given transmural vascular pressure is considerably greater in the former than the latter. Both are subjected to Pab which is assumed to act at the outer surface of all splanchnic blood vessels.

It is important to understand that at constant splanchnic blood volume, and vascular smooth muscle tone, a change in Pab imposes an equal change in intravascular pressure (Piv,sp). The transmural pressure (Ptm) across the splanchnic blood vessels (Piv,sp-Pab) is determined by splanchnic blood volume (Vsp) and the compliance of the splanchnic vasculature (Csp). Vsp is the sum of two components: the unstressed volume (Vus) and the stressed volume (Vs): Vsp = Vus+Vs. Vus is the volume of blood contained in the splanchnic vasculature when the Ptm is everywhere zero; Vs is the Vsp minus Vus. For Vs >0, Piv,sp > Pab and the splanchnic vasculature is distended by a positive Ptm. When this is the case, compression of the inferior vena cava (IVC) by positive Pab cannot stop blood flow as has been assumed [16]–[18]. If this were to happen, as soon as flow stopped the pressure just upstream from the obstruction would immediately rise to Piv,sp and the vein would reopen. Csp is given by the change in Vs per unit change in Ptm. Here the appropriate Piv,sp is the pressure in the splanchnic vasculature when all blood flow ceases, or the mean circulatory pressure (Pmc): Csp = ΔVs/Δ(Pmc-Pab) or the slope of the splanchnic vascular pressure volume curve at positive Ptm. For purposes of the present discussion we assume that no reflexes play a role in the phenomena we describe. Therefore at a constant Vsp, there can be no change in Ptm and any changes in Pab result in equal changes in Piv,sp.

We believe decreasing flow with increasing Pab shown in fig. 2-D, results from compression of the IVC resulting in a vascular waterfall [19]. The pressure driving flow out of the splanchnic vasculature into the IVC is Pmc relative to the pressure in the inferior vena cava (Pivc) where the hepatic vein empties into it [20]. The effective Ptm across the vasculature at the upstream end is Pmc–Pab, whereas at the downstream end it is Pivc-Pab. Initially Pab is less than both Pmc and Pivc and Ptm is positive throughout the vasculature [9]. It is important to remember that Pmc-Pab is the elastic recoil pressure of the splanchnic vasculature which is determined solely by Vs and Csp. In the absence of reflexes which change Csp, the elastic recoil pressure is constant at constant Vs, but when Pab increases and blood flows out of the splanchnic bed, Vs and the elastic recoil pressure both decrease. Nevertheless as long as Pivc > Pab, flow is proportional to Pmc– Pivc. This accounts for the flow regime where splanchnic outflow increases with Pab. But as soon as Pab exceeds Pivc the inferior vena cava becomes compressed and a vascular waterfall develops which limits splanchnic venous outflow.

Permutt and colleagues [19], [21], [22] have investigated this phenomenon extensively. They model it as a collapsible tube traversing a chamber with the inflow pressure, Pmc, greater than chamber pressure, Pab, which in turn is greater than the outflow pressure, Pivc. Under these conditions the effective pressure producing flow is the elastic recoil pressure of the splanchnic bed, and splanchnic flow is given by the product of the recoil pressure and the resistance of the uncompressed segment of vasculature [19]. This segment extends from the point in the vasculature where the pressure is Pmc at the inlet to the IVC where Ptm is zero. As perivascular pressure is Pab, the intravascular pressure is also Pab. This is called an equal pressure point [23] and the driving pressure from the inlet to this point is the elastic recoil pressure. As Pab continues to increase and Vs decreases the elastic recoil pressure must decrease and flow will decrease with it. This explains the regime where splanchnic outflow decreases with increases in Pab. If the elastic recoil pressure falls to zero, flow ceases, even though blood remains in the splanchnic bed as the unstressed volume.

Thus there are three potential pressure flow regimes in the splanchnic bed [24], [25]: 1) so-called zone 3 conditions where flow is dependent on Pmc– Pivc and the resistance from the small splanchnic veins to the inferior vena cava; here flow increases with Pab; 2) so-called zone 2 conditions where flow is dependent on the elastic recoil pressure of the splanchnic vasculature and the resistance of the uncompressed splanchnic venous bed down to the equal pressure point in the IVC; here flow decreases with increases in Pab due to the decrease in elastic recoil pressure and; 3) so-called zone 1 conditions where there is no flow because the stressed volume of splanchnic blood is zero and Piv,sp becomes less than Pab.

The first two flow regimes previously demonstrated in dogs [25], [26], also occur in humans. Furthermore we show that zone 1 conditions can exist when the stressed volume of the splanchnic bed falls to zero.

### Effects of Splanchnic Outflow on Cardiac Output

The relationship between splanchnic outflow and cardiac output depends upon interactions between the pressure where the hepatic vein enters the inferior vena cava and the venous return below this point. It is known that increases in Pab with breathing during exercise cause femoral venous flow to cease [16], [17]. This happens because the increased splanchnic outflow increases Pivc to the point where the energy gradient between the femoral and hepatic veins falls to zero. When this occurs all the inferior vena caval blood entering the right atrium will come from the splanchnic reservoir and blood will immediately pool in the legs. This provides the signal that we measure as Vbs. As total blood volume is constant this signal will equal the volume of blood displaced from the splanchnic bed and will be in phase with Pab. Preliminary experiments with an echo-Doppler probe show that femoral venous return falls to the extent that the probe can no longer detect any flow when Pab increases by 2–3 cm H_2_O. However flow restarts at substantially higher values of Pab due to pooling of blood in the legs (unpublished data).

As splanchnic outflow and Pivc begin to increase, flow below the hepatic vein will decrease and cardiac output should only change to the extent that the resistance from the hepatic vein to the right atrium is lower than that between the femoral vein and right atrium. When venous return from the lower extremities ceases the effect on cardiac output will depend on whether splanchnic venous flow is under zone 3 or zone 2 conditions. If the former is the case, cardiac output should increase whereas if the latter pertains cardiac output will decrease. Regardless, the increase in non-splanchnic systemic blood volume will increase the mean systemic vascular pressure by the volume of blood transferred divided by the compliance of the non-splanchnic vascular bed in the tissues drained by the IVC below the hepatic vein. This will increase venous return and cardiac ouput transiently once Pab decreases and the pressure gradient between the femoral vein and Pivc is restored.

The fact that Vbs could be as much as 650 ml (fig. 2-A) suggests that the stressed blood volume of the splanchnic vascular bed in humans is about equal to the unstressed volume. This is larger than generally accepted estimates of total blood stressed volume by Greenway and Lautt to be ∼40% of total blood volume [9]. However according to Rothe, in humans “the liver is the most important controllable blood reservoir in the body” [8]. Assuming that most of the hepatic blood is in the sinusoids and post-sinusoidal hepatic venules, the average vascular distending pressure should be close to that in the sinusoids. In the rabbit with the abdomen open and Pab = 0, this pressure averaged 5.4 mm Hg (0.6 kPa) greater than the pressure in the right atrium [15]. From this it is possible to estimate the hepatic stressed volume if the compliance of the hepatic vascular bed is known.

At a minimum the transmural or elastic recoil pressure in the sinusoids should be at least 5.4 mm Hg assuming atmospheric pressure to be the perivascular pressure. If atmospheric pressure were less than right atrial pressure, the elastic recoil pressure would be greater than 5.4 mm Hg. Risoe et al measured the splanchnic vascular capacitance in dogs [27]. From their data it is possible to estimate hepatic vascular compliance (Csp,h). When portal and hepatic venous pressures increased by 5.1 (0.6 kPa) and 7.3 mm Hg (1 kPa) respectively hepatic vascular volume rose by 6.3 ml*kg^−1^ of body weight. Assuming that the average increase in hepatic vascular pressure was the mean of 5.1 and 7.3 or 6.2 mm Hg (0.8 kPa), hepatic vascular compliance would be ∼1ml*mm Hg^−1^(7.5 ml*kPa^−1^). To the extent that values in rabbits and dogs are applicable to humans, a 70 kg person should have a Csp,h of 70 ml*cm H_2_O^−1^ (71.4ml*kPa^−1^). Therefore with a hepatic sinusoidal pressure of 5.4 mm Hg the stressed volume would be 378 ml.

This assumes that the hepatic vascular pressure volume curve is linear down to zero transmural pressure. Certainly vascular compliance increases as transmural pressure approaches zero (see figure 1 in ref 7). In fact, the hepatic stressed volume is probably greater than 400 ml because of the non-linearity of the hepatic vascular pressure volume curve, and because the transmural pressure in the sinusoids is probably greater than 5.4 mm Hg above ambient pressure because right atrial pressure is somewhat greater than atmospheric pressure. This estimate of hepatic stressed volume is in reasonable agreement with the volume leaving from the fast emptying compartment of the splanchnic bed shown by figures 1-C and 1-D.

If the volume of the fast space could be emptied during exercise this should increase the mean systemic vascular pressure, and would constitute a form of physiologic blood doping. This will increase perfusion of locomotor muscles and brain whose needs may be greater than those of the abdominal contents. A reduction in splanchnic blood volume has been reported during exercise [28], [29], when abdominal muscles are recruited and abdominal pressure increases [30]. As discussed below, this has important implications for exercise performance and cardiopulmonary resuscitation.

### Timing of Splanchnic Filling and Emptying

The time it takes to transfer blood to and from the splanchnic vascular bed depends on the mechanical properties of the splanchnic vasculature. Furthermore Piv,sp-Pab in the liver is somewhat less than Piv,sp-Pab in the non-hepatic vessels in order for blood to flow from the non-hepatic vessels through the portal vein to the liver [15]. This assumes that Pab is the same in both locations.

The time constant for filling of the non-hepatic bed (τsp,nh) is given by the product of the three resistances of the celiac, and superior and inferior mesenteric arteries arranged in parallel and the compliance of the non-hepatic vascular bed (Csp,nh). The resistance of the three arteries in parallel is given by 1/(Gc+Gsm+Gim) where Gc, Gsm and Gim are the conductances of the celiac, superior and inferior mesenteric arteries respectively. Thus τsp,nh = Csp,nh*1/(Gc+Gsm+Gim). The time constant for filling of the hepatic vasculature (τsp,h) is similarly given by Csp,h*1/(Gpv+Gha) where Gpv and Gha are the conductances of the portal vein and hepatic artery respectively. The fact that splanchnic vascular filling was well fitted by a single exponential (table 1) suggests that τsp,nh = τsp,h and Csp,nh*1/(Gc+Gsm+Gim) = Csp,h*1/(Gpv+Gha). If 1/(Gc+Gsm+Gim)<1/(Gpv+Gha), as seems likely Csp,h < Csp,nh. This is implied in hydraulic model shown in fig. 4 by the larger capacitance of the non-hepatic vascular bed.

The time constant for emptying the liver vasculature (τ'sp,h) is given by: τ'sp,h = Csp,h*Rhv, where Rhv is the resistance of the hepatic vein. Rhv is likely to be small because the vein is short. We found that is was nearly the same as the filling time constant. Therefore Rhv should be nearly equal to 1/(Gpv+Gha). We found that with step increases in Pab early emptying of the splanchnic bed was rapid and this was followed by slower emptying. We attribute the early phase to blood coming from the liver. Emptying of non-hepatic vessels however, is likely to be prolonged. The time constant for transferring blood from these vessels to the liver (τ'sp,nh) is given by: τ'sp,nh = Csp,nh*Rpv. As Rpv is almost certainly greater than the resistance of the arterial inflow to the non-hepatic vascular bed this emptying time constant will be longer than the filling time constant. Before this blood empties into the inferior vena cava, however, the time constant for filling of the liver vessels and the time constant for hepatic vascular emptying must be added to τ'sp,nh . With early emptying of hepatic blood and delayed refilling from non-hepatic blood the reduced hepatic blood volume will decrease the dimensions of the hepatic vasculature and thereby increase their resistance. It is not surprising that there appears to be a fast emptying compartment and a slower one, presumably hydraulically in series with the fast one as shown in table 1, figure 1-C and the hydraulic model in figure 4. Our findings that rapid, followed by slower exponential emptying from two compartment, accurately describes the experimental data (mean r^2^ = 0.983±0.01SD), support the hydraulic model's validity.

### Electrical Analogue and Practical Implications

**Figure 5:**
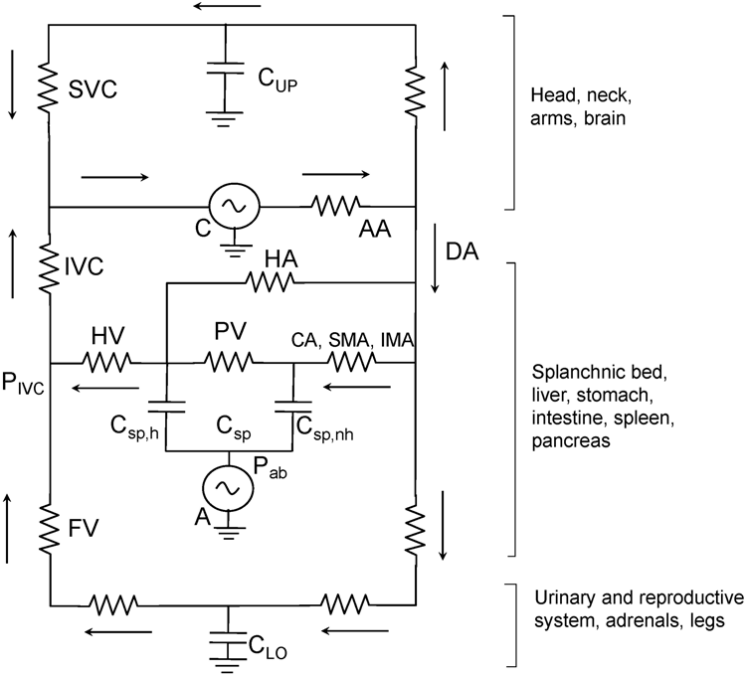
Electrical analogue of the circulatory system powered by two generators the cardiac pump (C) and the abdominal circulatory pump (A). Arrows indicate direction of blood flow. AA = aortic arch; DA = descending aorta; CA = celiac artery; SMA = superior mesenteric artery; IMA = inferior mesenteric artery; HA = hepatic artery; PV = portal vein; HV = hepatic vein; SVC = superior vena cava; IVC = inferior vena cava; FV = femoral vein; Pab = abdominal pressure; Pivc = pressure in the IVC at the point where the hepatic vein enters; Cup = compliance of capacitance vessels in the head and upper extremities; Clo = compliance of the capacitance vessels in the urinary and reproductive systems, the adrenal glands and the lower extremities; Csp = compliance of capacitance vessels in the splanchnic circulation, divided into two component compliances: Chep = compliance of the hepatic capacitance vessels and; Cnon-hep = compliance of the capacitance vessels of the non-hepatic abdominal viscera including stomach, intestines, spleen and pancreas. The generator C includes both left and right heart, the lungs and pulmonary circulation. For further description, see text.

Figure 5 is an electrical analogue of the circulatory system, powered by 2 generators. The one labeled ‘C’ represents the heart an involuntary pump while the one labeled ‘A’ represents the quasi-voluntary abdominal circulatory pump which controls the splanchnic blood reservoir. The cardiac pump accepts the whole venous return and pumps out an equal cardiac output which is divided into the amount perfusing the head, neck and arms, the splanchnic vasculature, and the flow to the lower extremities, adrenal glands, urinary tract and reproductive organs, Each of these three perfused regions contain capacitance vessels where blood can pool represented by the three capacitors, Cup for the upper extremities, Clo for the lower extremities and Csp for the splanchnic vascular bed. The last is divided into two, Csp,h representing the capacitance vessels of the liver and Csp,nh representing the capacitance vessels in the non-hepatic abdominal viscera.

In the absence of both pumps no blood would circulate and the intravascular pressures (neglecting hydrostatic effects) would become Pmc. Blood would pool in Cup, Clo and Csp, by an amount determined by Pmc and the respective compliances of each capacitor. Cup and Clo are grounded, i.e., the extravascular pressure is atmospheric, but the extravascular pressure for Csp is Pab. Pmc is less than mean arterial pressure and greater than right atrial pressure [8], [20] and is thought to be about 10 cm H_2_O (1 kPa).

When the heart, C, is beating, there will be unique sites in all three circulatory beds where the intravascular pressure equals Pmc, These sites are represented in the analogue where the capacitors are situated in each circulatory pathway, Then the driving pressure for venous return according to Guyton's concepts is Pmc for each bed [20] . The venous return from the inferior vena cava (IVC) is the sum of the venous return coming from the lower extremities and the splanchnic circulation.

If both C and A are active the abdominal pump removes blood from the reservoir and increases the amount of blood entering the IVC through the hepatic vein. As discussed above, this in turn increases the pressure in the IVC where the hepatic vein enters and decreases the energy difference between the capacitance vessels represented by Clo, and the IVC thereby decreasing the blood flow from the lower extremities and increasing the blood volume stored in Clo. If the abdominal circulatory pump is sufficiently active the pressure difference between Clo and IVC may fall to zero and all venous return from the lower extremities will cease as we have found during small increases in Pab (unpublished data) and Miller et al [16], [17] have reported during exercise. When femoral venous flow ceases even more blood will be stored in Clo increasing the intravascular pressure at Clo by an amount equal to the increase in blood volume divided by the compliance of the lower extremity capacitance vessels. As a result when Pab falls venous return from the legs should increase. If this were to happen during exercise the physiologic blood doping provided by Vbs would increase the amount of energy delivered to working locomotor muscles. As stated above we found blood shifts in the order of 300 ml during exercise in normal subjects, but only when abdominal pressures became unusually high resulting from an imposed expiratory flow-limitation [1], [31]. Little or no blood shifts were detectable during control exercise, even though it is known that abdominal muscles are recruited during expiration at the onset of even mild exercise [30]. Whether exercise performance can be improved by greater voluntary abdominal muscle contraction needs to be investigated.

**Figure 6:**
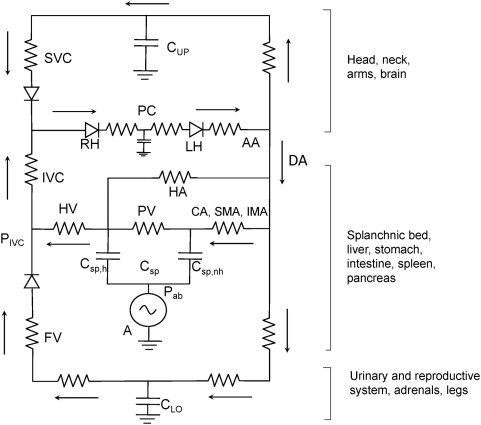
Electrical analogue of asystolic cardiac arrest . The symbols are the same as in figure 5 with the addition of RH = right heart; LH = left heart, both of which are now represented by diodes, and PC = pulmonary circulation. Diodes in the femoral vein and superior vena cava represent venous valves that ensure that blood entering the inferior vena cava flows through the heart and pulmonary circulation into the aorta. For further description, see text.

To the extent that the electrical analogue represents reality, what might be expected of the abdominal circulatory pump if C were inactive due to asystolic cardiac arrest? This is shown in figure 6 the electrical analogue of figure 5 where the heart has been replaced by two diodes one representing the right heart and the other the left heart with the pulmonary circulation in between. With A inactive, blood pressure would immediately fall to Pmc. However when A starts pumping with increases in abdominal pressure, the intravascular pressure generated by A in the splanchnic vasculature would become the point of highest pressure in the circulatory system. Diodes in the superior vena cava and femoral vein represent venous valves that ensure that the blood would flow from the splanchnic bed into the IVC, right atrium, through the pulmonary circulation and the heart into the aorta and perfuse systemic body tissues. This flow would have to be sufficient to keep the brain and other vital organs adequately perfused. We know that coughing during asystolic cardiac arrest can maintain consciousness [4], [5] so this scenario seems realistic.

**Figure 7:**
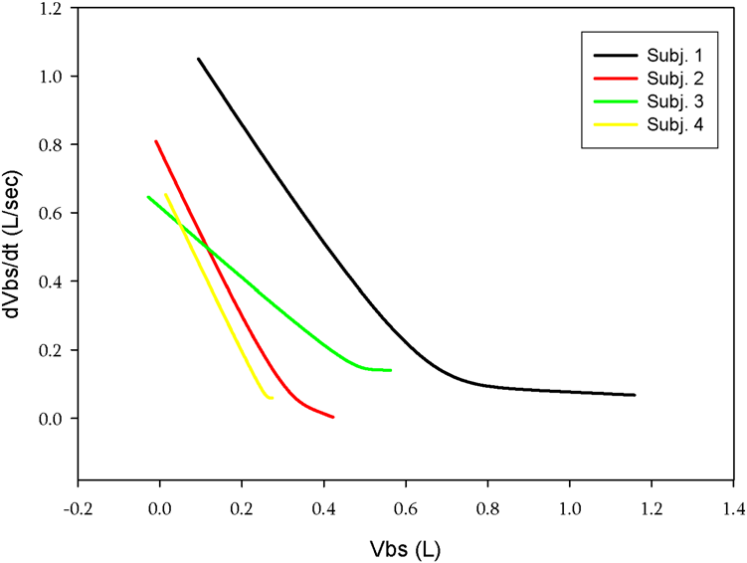
Relationship between splanchnic outflow and splanchnic blood volume following a step increase in Pab lasting for ∼10 sec. in four male subjects. Each line pattern represents a different subject. In all subjects there is initial, exponential emptying of a fast compartment, with a steep and linear slope equal to the rate constant of emptying, followed by a slower compartment and a longer rate constant.

For optimal perfusion, when only the abdominal pump is functioning, blood flow would ideally be the maximal possible under zone 3 conditions and the amount of blood in the splanchnic reservoir should be large prior to each ‘systole’. By differentiating the volume of blood displaced during a large step increase in Pab as a function of time shown in fig. 1-C we obtain the maximal splanchnic outflow. When this is plotted against the volume of splanchnic blood expelled we obtain the maximal splanchnic outflow as a function of splanchnic blood volume. This is shown in figure 7 and is analogous to the maximal expiratory flow-volume curve of the lung [32]. The initial part of the curve is dominated by emptying of the fast space while the latter part represents emptying of the slow space. Emptying of the fast space provides a ‘stroke’ volume of ∼300 ml (also illustrated in fig. 1-C and fig. 1-D). The short filling time constant we measured indicates that following such a stroke volume the splanchnic reservoir can be adequately refilled in 2 seconds, prior to the next systole. Thus, the flow regime should remain in zone 3 conditions. Hence during asystolic cardiac arrest, a step increase in Pab of ∼100 cm H_2_O lasting 1 second followed by a diastole of 2 seconds and thus a duty cycle of 0.33 should result in a stroke volume of 300 ml which repeated at a frequency of 20/min would result in a splanchnic output of 6L/min. We believe this is might be feasible during cardiac arrest and that the abdominal circulatory pump might act as an auxiliary heart.

There are several reports of abdominal compression interposed with chest compression during cardiopulmonary resuscitation (IAC-CPR) in both humans and experimental animals. In experimental animals it increases carotid blood flow [33], arterial pressure [34], [35], cardiac output [34], coronary perfusion pressure and survival [35]. In humans it resulted in higher arterial pressures [36], [37], increased end-tidal partial pressures of CO_2_ suggesting an increased cardiac output [37] and, when performed in hospital, improved survival [38], [39]. However the difference between diastolic arterial and venous pressures (a measure of coronary perfusion pressures) were below the minimum needed for resuscitation in animal models [40] and out-of-hospital IAC-PCR did not improve survival [41]. There was no evidence that abdominal compression resulted in increased abdominal injury [36], [37]. These studies are difficult to evaluate because in most, the compression rate was considerably greater than 20/min, so that ‘diastolic’ refilling of the splanchnic bed was probably inadequate resulting in limited flow under zone 2 conditions coming from non-hepatic viscera with long emptying time constants. Knowledge of filling and emptying time constants under zone 3 conditions should improve the efficacy of abdominal compression during cardiopulmonary resuscitation.

To summarize, we present the first comprehensive study of the effects of abdominal pressure on the splanchnic vascular bed. A step increase in abdominal pressure resulted in initial rapid splanchnic emptying from a fast space, presumably the liver, followed by much slower emptying from non-hepatic abdominal viscera. Filling was well-described by a single exponential with a time constant closely similar to the emptying of the fast compartment. Ramp increases in abdominal pressure resulted in increases in splanchnic outflow, followed by decreases as abdominal pressure continued to increase. We attribute the reduction in flow to the development of a vascular waterfall resulting from compression of the inferior vena cava just rostral to its passage through the diaphragm by the high abdominal pressures. If so this would change the splanchnic flow from zone 3 to zone 2 conditions. As splanchnic outflow increases through the hepatic vein, the blood pressure where it enters the inferior vena cava will also increase thereby decreasing the pressure gradient producing venous return from the lower extremities, kidneys and urinary tract, adrenal glands and reproductive organs. In the lower extremities this resulted in the blood shifts we measured from the trunk to the extremities.

Recruitment of splanchnic blood to the extremities during exercise could increase perfusion to locomotor muscles and might act as physiologic blood doping to increase performance. During asystolic cardiac arrest, we calculate that in a normovolemic individual, with appropriate timing of abdominal ‘systole’ and ‘diastole’ the abdominal circulatory pump might act as an accessory heart to produce a normal resting circulatory output.

## Methods

### Ethical approval

The project was approved by the ethics committee of the INRCA Hospital. The subjects were trained laboratory personnel, experienced in respiratory maneuvers, who volunteered for the experiment. All gave informed consent after personal risks were explained and the benefits of the research were outlined. They were free to withdraw at any time without penalty. A physician was present at all experiments, and the studies conformed to the latest revision of the Declaration of Helsinki..

### Subjects and Measurements

The experiments were performed on seven healthy subjects, five males and two females aged between 25 and 40 years. Complete data were only obtained in five. They sat in a homemade, transparent, variable flow, whole body plethysmograph which measured ΔVb while breathing room air through a mouthpiece connected to a pneumotachygraph mounted outside the plethysmograph (fig. 3-A). ΔVtr was measured by OEP [2]. Eighty-nine reflective markers were attached to the trunk from clavicles to pubis anteriorly and posteriorly (fig. 3-B). Each marker was tracked continuously by video-cameras outside the box and Vtr calculated by Gauss's theorem [2]. Balloon catheters in the stomach and esophagus attached to piezoresistive transducers (ASDX005D44D-A, full range scale ±351 cmH_2_O, Sensortechnics Munich, Germany) measured abdominal and pleural pressure (Pab and Ppl) respectively. Pressure at the mouth (Pm), equal to alveolar pressure at zero flow with the glottis open, was measured by a catheter connecting the mouthpiece to another identical piezoresistive pressure transducer.

### Double Plethsmography

**Figure 8:**
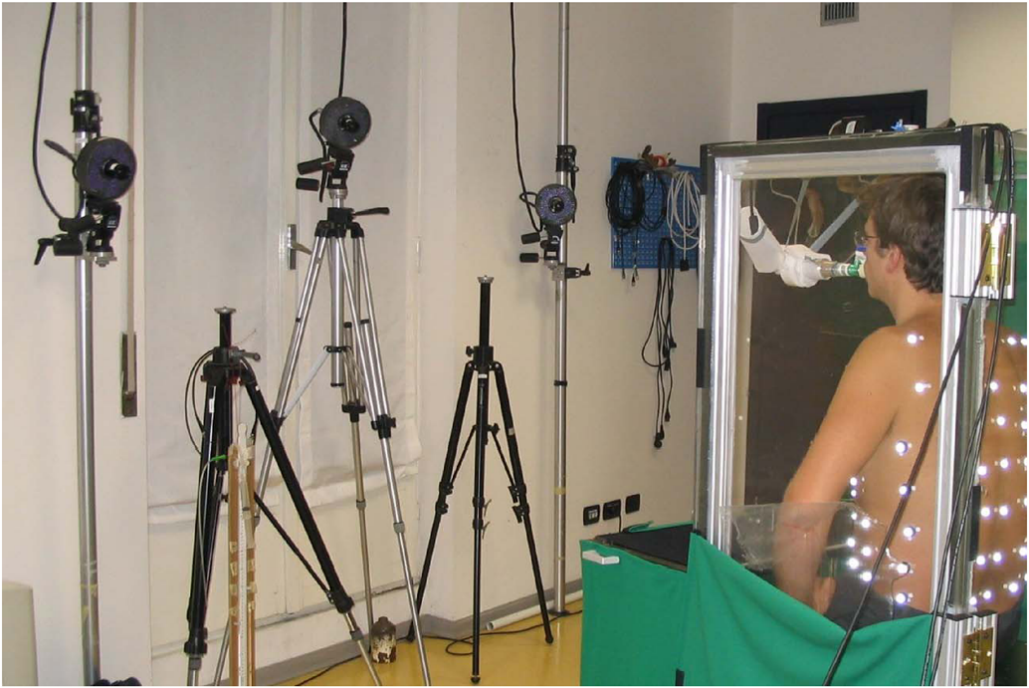
Photograph of a subject inside the transparent whole body plethysmograph with body surface markers for optoelectronic plethysmography in place. The cameras recording motion of the anterior markers are shown in the background.

The advance that made the present communication possible was the combination of whole body and optoelectronic plethysmography. The whole body plethysmograph was constructed out of plexiglass with a thickness of 20 mm. It was designed with two transparent flat walls in front of and behind the trunk of the subject under analysis. A photograph of a subject inside the box with markers on his trunk and some of the cameras used to track the markers for OEP is shown in figure 8.

In lateral view the body box was ‘L’ shaped with a base larger (89*cm×*72*cm* ) than the top to allow the positioning of the legs. The door was mounted on the lateral wall and sealed with closed cell sponge rubber to prevent leakage of air. The height and the volume of the box were 158 cm and 709 liters respectively. The subject sat on an ergonomic chair that kept him/her in a comfortable posture for long periods of data acquisition. The seat allowed the thighs to descend at a 45° angle and provided support for the knees. This extended the trunk allowing for better visibility of the markers placed on the lower abdomen by displacing the thighs downward away form the abdomen.

The body box was equipped with two linear mesh resistances (total resistance = 0.255 cmH_2_O·sec·L^−1^) mounted above the subject's head through the wall of the box, which were used to measure flow in and out of the box as the subject breathed room air through a mouthpiece mounted in the front wall of the box. The 3-D displacements of the 89 reflective markers were detected by three video cameras mounted in front and three behind the body box at a distance of about 2 meters. The optical distortion introduced by the presence of the two transparent plexiglass walls between the cameras and the markers was corrected by moving a bar holding markers placed at known distances within the box and then applying the usual algorithms for camera calibration (Thor2®, BTS, Milano, Italy). To demonstrate that they were valid we correctly measured solids of known volume placed inside the box. The measured error of the measurements was always <±0.3%.

The piezoresistive pressure transducers used to measure esophageal and gastric pressures were placed outside the body box and the catheters were attached to them through connections placed through the walls of the box.

Flow in and out of the body box was derived by measuring pressure variations within the box by a highly sensitive pressure sensor (PCLA02X5D, full range scale ±2.55 cmH_2_O, Sensortechnics, Munich, Germany). This is a miniaturized amplified output pressure sensor based upon a technology able to reduce all output offset or common mode errors due to change in temperature, and characterized by high stability over extended time periods.

Temperature within the box was continuously measured by a glass-encapsulated NTC (negative temperature coefficient) resistance sensor (B57550G502J, EPCOS AG, Munich, Germany).

The signal of the flow across the body box was anti-filtered in order to correct for the dynamics of the box itself. Body box dynamics were modelled by defining an electrical analogue model where electrical currents and voltages correspond to gas flow and pressure, respectively. A generator of electrical current (I) models total lung volume variations generated by the subject (dV_L_/dt ) breathing through the box, which lead in part to a flow of air through body box walls and in part to compression of gas contained in the box. A capacitor C represents the compressibility of the gas contained in the box (Vair), depending on the volume of the air surrounding the subject (Vair = Vbox – Vb, where Vbox is the 709L volume of the box and Vb = total body volume, approximately body weight, in Liters). We assumed that the capacitance of air in the body box was constant because changes in body volume with breathing are two orders of magnitude less than Vair. The corresponding impedance due to C is: Z_1_ = 1/sC. A resistance R (with an impedance Z_2_ = R) models the resistance of the pneumotachographs placed across the box to measure flow. In our model, I, C and R are all in parallel.

**Figure 9:**
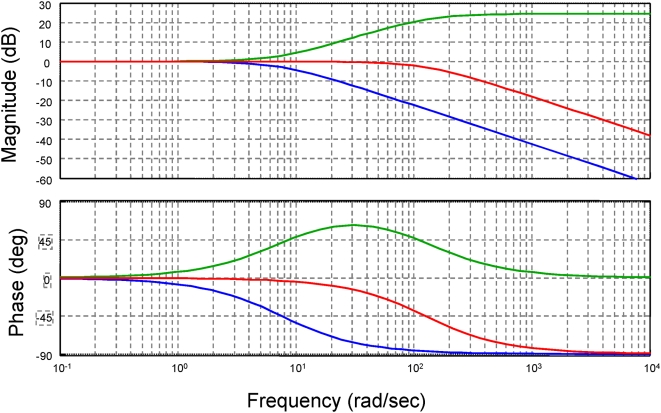
Frequency response of the variable flow whole body plethysmograph. Blue line is the box alone; green line is the antifilter alone; red line is the box and antifilter combined.

In this simple model, the flow through R is I_R_ = I/(1+sRC) and therefore the transfer function between lung volume variations and flow measured though the box is equivalent to a low-pass first order filter with a cut-off frequency fc = 1/2πRC = 1/2πτ_BOX_ . By applying a square wave of flow at the connection port of the mouthpiece and measuring the flow across the box, we estimated a cut-off frequency of 1.20 Hz, causing a significant delay. This delay was corrected by implementing an Infinite Impulse Response digital filter (Matlab: The Mathworks, Natick, MA) whose complete transfer function was:
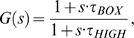
with f_HIGH_ = 1/2πτ_HIGH_ is the cut off frequency of the low pass filter used to eliminate high frequency noise. The frequency response of the system is shown in figure 9. That of the box alone is shown by the blue line, that of the antifilter by the green line and the box and antifilter combined by the red line.

### Procedures

Measurements were made during: 1) quiet breathing; 2) breathing with stronger diaphragmatic contractions and increasing Pab; 3) rib cage breathing in an attempt to keep Pab constant; 4) diaphragmatic breathing with inspiratory increases in Pab while contracting abdominal muscles during expiration to maintain a higher than normal Pab throughout the respiratory cycle. Subjects performed expulsive maneuvers to increase Pab by simultaneous contraction of the diaphragm and abdominal muscles while maintaining the glottis open and avoiding increases in Ppl. These expulsive maneuvers were performed as square waves with step changes in Pab, and as ramp increases. They avoided the complexities of Valsalva's maneuver.

### Data Analysis

Vbs was measured as ΔVb-ΔVtr during the various breathing patterns and during the expulsive maneuvers. The responses of Vbs to the step changes in Pab were fitted by first order exponentials, from which time constants for emptying and filling of the splanchnic bed were calculated. Two exponentials were required to reproduce the time course of emptying, whereas filling was well-fitted by a single exponential. The ramp increases in Pab gave the volume of splanchnic reservoir blood quasi-statically expelled per unit increase in Pab. After appropriate filtering and curve-fitting by a sigmoidal function, the slope of the Vbs time plot was used to obtain hepatic venous outflow as a function of Pab and time.

Heating and/or humidification of air inside the body plethysmograph or integrator drift appeared as an increase (or in the case of integrator drift, also a decrease) in lung volume with no change in Vtr and might be interpreted spuriously as a blood shift. However, under these circumstances, lung volume would appear to change with no change in transpulmonary pressure (measured as the difference between mouth and esophageal pressure). As transpulmonary pressure and lung volume are inextricably linked in normal subjects by the lungs' pressure volume relationship, the spurious nature of the apparent change in volume was revealed by the failure of transpulmonary pressure to change. When this occurred the data were either discarded or used to correct for drift, heating and/or humidification.

## Supporting Information

Appendix S1 List of abbreviations
(0.03 MB DOC)
