# Supplementary material for: The Abdominal Circulatory Pump
Source: PLoS One. 2009 May 14;4(5):e5550. doi: 10.1371/journal.pone.0005550 (PMC2678249; doi:10.1371/journal.pone.0005550)
Supplement: Appendix S1 — List of abbreviations (0.03 MB DOC) [file pone.0005550.s001.doc]

**Appendix S1**

***Glossary of symbols***

WBP: whole body plethysmography

OEP: opto-electronic plethysmography

IVC: inferior vena cava

PEEP: positive end-expiratory pressure

H: heart

A: abdominal circulatory pump

C: compliance; Csp: compliance of the splanchnic vasculature; Csp,h: compliance of the hepatic vasculature; Csp,nh: compliance of the non-hepatic splanchnic vasculature; Clo: compliance of the lower body vascular bed; Cup: compliance of the upper body vascular bed

G: conductance; Gc: conductance of the celiac artery; Gha: conductance of the hepatic artery; Gima: conductance of the inferior mesenteric artery; Gpv: conductance of the portal vein; Gsma: conductance of the superior mesenteric artery

P: pressure; Pab: abdominal pressure; Pivc: pressure in the inferior vena cava; Piv,sp: intravascular pressure in the splanchnic bed; Pmc: mean circulatory pressure; Ppl: pleural pressure; Ptm: vascular transmural pressure

**τ**: time constant; **τ**sp,h; blood filling time constant of the liver; **τ**sp,nh: blood filling time constant of the non-hepatic abdominal viscera; **τ’**sp,h: blood emptying time constant of the liver; **τ’**sp,nh: blood emptying time constant of the non-hepatic abdominal viscera

R: Resistance; Rhv: resistance of the hepatic vein; Rpv: resistance of the portal vein

V: volume; Vb: volume of the body; Vtr: volume of the trunk; Vbs: volume of blood shifts; VL: lung volume; Vcom: volume of gas compressed within the lung; Vsp: splanchnic blood volume; Vs: stressed blood volume; Vus: unstressed blood volume.

IAC-CPR: Interposed abdominal compression cardiopulmonary resuscitation.

Some symbols that appear only in the figures are explained in the figure legend
